# Evaluation of CD10 expression as a diagnostic marker for colorectal cancer 

**Published:** 2022

**Authors:** Jakub Żurawski, Patrycja Talarska, Mateusz de Mezer, Krzysztof Kaszkowiak, Michał Chalcarz, Katarzyna Iwanik, Jacek Karoń, Piotr Krokowicz

**Affiliations:** 1 *Department of Immunobiology, Poznan University of Medical Sciences, Poland*; 2 *Chalcarz Clinic, Aesthetic Surgery, Aesthetic Medicine, Polan*; 3 *Chair and Department of Clinical and Immunological Pathology, Poznan University of Medical Sciences, Poland*; 4 *Clinic of General and Colorectal Surgery, Poznan University of Medical Sciences, Poland*

**Keywords:** CD10, Colorectal cancer, Immunohistochemistry, ELISA

## Abstract

**Aim:**

We aimed to determine the potential of CD10 as a marker for the early diagnosis of adenocarcinoma of the colon.

**Background:**

Adenocarcinoma is diagnosed in one out of 20 individuals in the USA and western European countries. Its prognosis and treatment depend largely on the severity of the disease at the time of diagnosis. Additional new biological markers are being sought that can help diagnose colon cancer at an early stage. One such marker present in both serum and tumor tissue is CD10.

**Methods:**

CD10 concentrations were tested by ELISA and immunohistochemistry in serum and tissue samples, respectively, from 113 patients diagnosed histopathologically and treated for adenocarcinoma of the colon. Additionally, the ROC curve with optimal cut-off point based on Youden’s criterion was calculated for CD10.

**Results:**

Serum concentrations of CD10 and its tissue expression in patients diagnosed with adenocarcinoma of the colon correlate with cancer staging based on the Astler-Coller-Dukes classification. To ascertain the optimal cut-off point for CD10 as a predictor of belonging to the study group, ROC curve was prepared for CD10. Optimal cut-off point for CD10 was 0.57, with prediction of belonging to the study group for CD10 ≥ 0.57.

**Conclusion:**

CD10 can be a useful marker in the early diagnosis of adenocarcinoma of the colon

## Introduction

 The high availability of endoscopic screening tests and the knowledge that most cancers originate from adenomas make it possible to prevent the development of malignant tumors of the large intestine. Unfortunately, colorectal cancers develop latently, asymptomatically, and may be undetectable for a long time.

Adenocarcinoma of the colon is diagnosed in one out of 20 individuals in the USA and western European countries. It accounts for 15% of all solid tumors diagnosed in developed countries. Its prognosis and treatment depend, to a large extent, on how advanced the disease is at the time of diagnosis. More than 70% of cases are diagnosed at an early clinical stage, enabling effective, radical surgical treatment. Unfortunately, about 20% of the cases with metastases at the time of diagnosis have a poor prognosis (1).

There is a definite need for additional new biological markers that can help diagnose colon cancer at an early stage. One such marker, present both in the serum and tumor tissue, is CD10. Significantly, it is not present in the regular mucous membrane of the colon, but it may be present in its epithelium and stroma. Additionally, it is not present in adenocarcinomas with low-grade dysplasia but is present in those with high-grade dysplasia (2).

The incidence of colon cancer varies worldwide, with almost 55% of cases occurring in highly developed countries. The survival rates for patients with colorectal cancer also vary from country to country. While the 5-year survival rate in the US is 65% (3), it ranges from 27% to 85% in Iran (4), which is lower than in other developed countries.

CD10 (neprilysin, CALLA – common acute lymphoblastic leukemia antigen, MME - membrane metalloendopeptidase, neutral endopeptidase, enkephalinase) belongs to the group of transmembrane metalloproteinases with zinc-dependent endopeptidase activity. It is involved in the degradation of various peptides on the amino side of hydrophobic residue and several peptide hormones. It is coded by the MME gene, which is located on chromosome 3 (5).

CD10 is expressed in immature cells of precursor T, B lymphocytes as well as in dividing B cells and mature neutrophils. It is also found in the kidneys, intestine, liver, and endometrium (6, 7). CD10 is also a marker for the diagnosis of acute lymphoblastic leukemia, B-cell lymphoma, and sporadically mantle cell lymphoma and stromal cells in invasive mammary carcinomas (8).

Increased tissue expression levels of CD10 vary depending on the tissue and stage of the disease. CD10 expression has been reported in breast, lung, and colon cancers, adenocarcinomas of the prostate and pancreas, and malignant melanomas. It is also found in malignant carcinomas and has been described as a predictor of aggressive cancer as a result of extracellular enzymatic degradation and altered signaling (9).

CD10 expression in advanced colorectal cancer is associated with the development of liver metastases. Therefore, determining CD10 expression early can enable intensive treatment and a better prognosis (10). However, the literature on CD10 as a potential diagnostic marker for colorectal adenocarcinoma is limited. Moreover, no studies have assessed CD10 levels in the serum and tissue of colorectal cancer patients in relation to the degree of histological malignancy.

The current study purposed to evaluate the serum and tissue concentrations of CD10 and their association with cancer stage based on the Astler-Coller-Dukes classification in patients diagnosed with adenocarcinoma of the colon. We aimed to determine the potential of CD10 as a marker for the early diagnosis of adenocarcinoma of the colon. 

## Methods

**Study participants**

Serum CD10 concentration was determined by enzyme-linked immunosorbent assay (ELISA) and immunohistochemistry. Tumor samples collected during surgery from 113 patients diagnosed with colon adenocarcinoma based on histopathology were tested for CD10 levels. Out of 387 patients who initially qualified for the study, 274 with a medical history of coexisting inflammatory diseases (ulcerative colitis, Crohn's disease) and other neoplastic diseases were excluded.

The control group included 20 patients who qualified for an appendectomy. For ethical reasons, no healthy tissue was collected for research. Therefore, only serum levels of CD10 were determined in the control group, and no immunohistochemical tests on tissue samples were performed. Medical surveys, biochemical and radiological examinations did not show any carcinomas in these patients.

Informed consent to participate in the study was obtained from all patients. The study was approved by the local Bioethics Committee.

**Tissue Immunohistochemistry**

Formalin‐fixed and paraffin‐embedded blocks were cut into 5‐μm thick slices. After deparaffinization, rehydration, blocking of endogenous peroxidase, and antigen retrieval, the sections were exposed to anti-CD10 (clone 56C6; Novocastra, UK; diluted 1:100) monoclonal antibodies. A polymer‐based detection system (Envision+; Dako, Carpinteria, CA, USA) with hematoxylin counterstaining was used. Sections treated with matched concentrations of non-immune IgG were used as a negative control. Lymph nodes (germinal center from the lymph node cortex) were a positive control for immunohistochemical staining.

The immunohistochemical reaction was evaluated semi-quantitatively using the commercial Olympus cellSens dimension program on the Olympus BX 43 light microscope and an XC 30 digital camera (Olympus, Tokyo, Japan). Phase analysis of immunohistochemically stained histological slides was performed using the software, including automatic detection of objects based on color, shade intensity, or shape. In this case, the color criterion (brown chromogen 3,3'-diaminobenzidine) was chosen. The program automatically classified the cells and stained areas based on predefined threshold values. The data was exported to MS Excel files used for further statistical analysis (11).

**ELISA with the commercial kit**

All serum samples were analyzed using a commercially available Human Neprilysin SimpleStep ELISA^®^ kit (Abcam catalog number: ab234563) as per the manufacturer's guidelines. The optical density of the reaction products was measured using an ELISA reader (EPOCH, BioTek). The detection range for the assay was 156-10000 pg/ml with a sensitivity of 97.2 pg/ml. Duplicate readings for each standard, control, and sample were averaged, and the background (zero standard) was subtracted. The concentrations in the samples were determined based on the standard curve plotted for each test run.

**Histopathological examination**

Colorectal cancer staging was based on the Astler-Coller-Dukes classification. Six stages A, B1, B2, C1, C2, and D, were defined based on the extent of metastasis (12).

**Statistical analysis**

Statistical analysis was performed using R-3.5.1 software. Descriptive statistics were used to present the examined variables. The normality of distribution of the quantitative variables was estimated using the Shapiro-Wilk test, skewness, and data kurtosis as well as visual evaluation of histograms. Homogeneity of variance was tested using Levene’s test. The groups were compared using the Chi-square or Student’s t-student test, depending on the situation. Due to deviating observations and the ordinal character of data for the Astler-Coller-Dukes scale, dependencies between variables of CD10 concentration in the tissue and the Astler-Coller-Dukes scale were verified using the Spearman correlation coefficient. All tests were two-sided, and a significance level of α = 0.05 was used.

Additionally, the ROC curve with an optimal cut-off point based on Youden’s criterion was calculated for CD10 as a discriminator of the study group (patients diagnosed with colorectal adenocarcinoma based on histopathological examination and treated) and the control group (blood serum collected from patients who qualified for appendectomy). 

## Results

The current study included 113 patients (68 women (60%) and 45 men (40%)) with adenocarcinoma of the colon diagnosed on the basis of histopathological examination. The control group comprised 20 patients (10 women and 10 men). No significant difference was seen in terms of gender between the two groups. 

While the median age of the study group was 59.02 ± 10.16 years, that of the control group was 35.25 ± 9.71 years (*p* < 0.001) (Table 1).

**Table 1:** Demographic characteristics of the patients

	Study group	Control group	*P*
*N*	113	20	
Age (years)	59.02 ± 10.16	35.25 ± 9.71	< 0.001
Gender, *n *(%)			
Female	68 (60.2)	10 (50.0)	0.545
Male	45 (39.8)	10 (50.0)	

Data are presented as arithmetic mean ± standard deviation unless marked otherwise. The groups were compared using the Student’s t-test or chi-square test.

**Table 2:** Clinical characteristics of the patients

Variable	Colorectal cancer staging (Astler-Coller-Dukes)	Study group	Control group	*MD *(95% *CI*)	*P*
*n*		113	20		
Serum CD10 concetration		0.90 ± 0.28	0.51 ± 0.04	0.39 (0.33; 0.44)	< 0.001
CD10 tissue expression (µm^2^)		18.48 ± 11.16	-	-	n/d
Colorectal cancer staging (Astler-Coller-Dukes) *n* (%)	A	20 (17.7)	-	-	n/d
	B1	22 (19.5)	-	-	
	B2	24 (21.2)	-	-	
			
	C1	23 (20.4)	-	-	
	C2	24 (21.2)	-	-	

Data are presented as arithmetic mean ± standard deviation unless marked otherwise. *MD*: mean difference between groups with a 95% level of trust. Student’s test.

**Figure 1:**
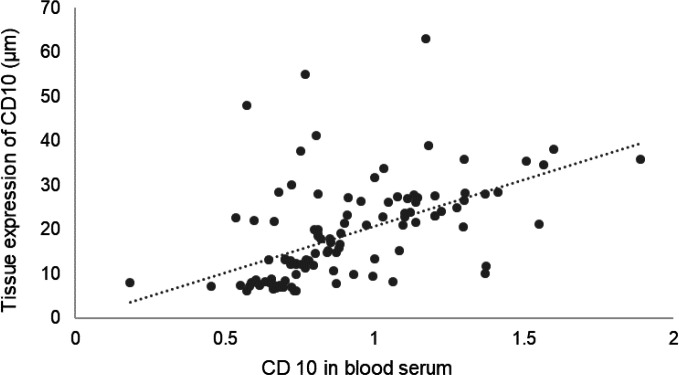
Scatter plot of CD10 concentration in serum and its tissue expression in the study group (*r*_s_ = 0.65, *p* < 0.001)

The mean serum concentrations of CD10 in the study and control groups were 0.90 ± 0.28, and 0.51 ± 0.04, respectively. The difference in the mean CD10 concentrations was statistically significant with mean difference (*MD)* = 0.39, *CI*_95_ (0.33; 0.44), *p* < 0.001.

The average area of CD10 immunohistochemical staining in the study group was 18.8 ± 11.16 µm^2^ (range: 6.02 µm^2^ - 62.89 µm^2^).

Colorectal cancer staging based on the Astler-Coller-Dukes classification showed a uniform distribution with a comparable number of patients in the different groups. Infiltration limited to the mucosa (stage A) was identified in 20 patients. Infiltration into the muscularis propria, with no penetration through it (stage B1), was identified in 22 patients, and penetration through the muscularis propria (stage B2) was identified in 24 patients. Involvement of regional lymph nodes with infiltration into the muscularis propria, but no penetration through it (stage C1), was identified in 23 patients, and the remaining 24 patients showed infiltration through the muscularis propria with penetration and involvement of lymph nodes (stage C2) (Table 2).

**Figure 2:**
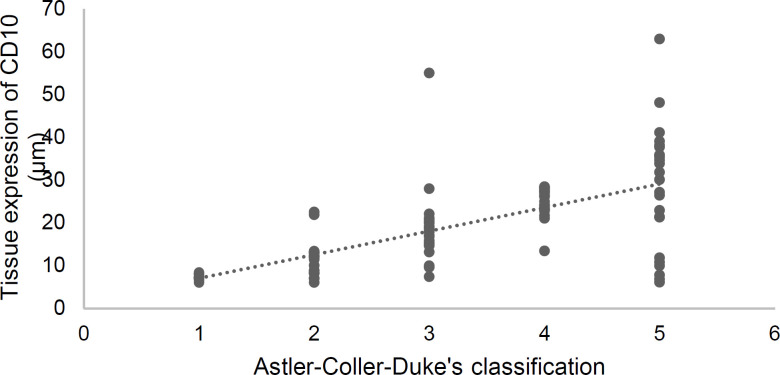
Scatter plot of CD10 tissue expression based on the Astler-Coller-Dukes classification of colon cancer in the study group (*r*_s_ = 0.74; *p* < 0.001)

**Figure 3:**
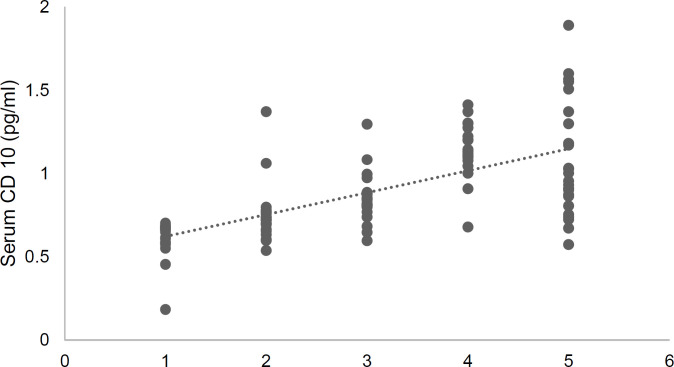
Scatter plot of CD10 serum concetration based on the Astler-Coller-Dukes classification of colon cancer in the study group (*r*_s_ = 0.70; *p* < 0.001)

In the study group, a positive correlation was seen between the serum CD10 concentration and the area of CD10 immunohistochemical staining in the corresponding tissue samples (*r*_s_ = 0.65, *p* < 0.001) (Table 3, Figures 1-3).

Both serum and tissue levels of CD10 showed significant positive correlations with the Astler-Coller-Dukes classification (*r*_s_ = 0.70; *p* < 0.001 and *r*_s_ = 0.74; *p* < 0.001, respectively). These findings suggest that advanced stages of cancer were associated with both higher serum concentration and tissue expression of CD10. Figures 1-3 present these correlations.

Astler-Coller-Dukes classification: 1-A; 2-B1; 3-B2; 4-C1; and 5-C2

Astler-Coller-Dukes classification: 1-A; 2-B1; 3-B2; 4-C1; and 5-C2

**Table 3:** Correlation between concentration of CD10, its tissue expression, and the Astler-Coller-Dukes scale in the study group

Correlation	Rho correlation coefficient	*P*
Serum concetrantion and tissue expression (µm^2^) of CD10	0.65	< 0.001
Astler-Coller-Dukes scale and serum concetration of CD 10	0.70	< 0.001
Astler-Coller-Dukes scale and tissue expression of CD10 (µm^2^)	0.74	< 0.001

To ascertain the optimal cut-off point for CD10 as a predictor of belonging to the study group,* ROC* curve was prepared for CD10. The analysis resulted in the area under the curve *AUC* = 0.979, *CI*_95_ (0.954; 1.0003), meaning a very high differentiation of patients based on CD10. The optimal cut-off point for CD10 was 0.57, with prediction of belonging to the study group at CD10 ≥ 0.57. Sensitivity of the analysis was 96% and specificity was 100%, meaning that 96% of patients from the study group and 85% of patients from the control group were correctly identified using the determined CD10 cut-off point. 

Accuracy of the test (*ACC*) was on the level of 97%, meaning 97% of all patients were correctly identified as members of the study or control group based on the CD10 cut-off point of 0.57.

## Discussion

Adenocarcinoma of the colon is the most common malignancy of the gastrointestinal tract. The peak incidence for colorectal carcinomas is put at 60-70 years of age, and it has been shown to occur more commonly in men than women. However, in the study group of 113 patients with a median age of 59.02 ± 10.16, there were more women (68/113; 60%) compared to men (45; 40%). Poor histological differentiation and mucinous type of tumor tissue are associated with a poor prognosis. However, the most important prognostic factors are the extent of tumor infiltration and metastases to the lymph nodes (13).

In this study, while infiltration was limited to the mucous membrane in 20 patients, it extended to and penetrated through the muscularis propria in 46 patients. Metastasis to the lymph nodes was observed in 47 patients. Cancer staging based on the Astler-Coller-Dukes classification showed a significant correlation with CD10 concentration in both the serum (*r*_s_ = 0.70, *p* < 0.001) and tissue (*r*_s_ = 0.74, *p* < 0.01). These results suggest that the proteolytic effects of CD10 expression are controlled by the surrounding microenvironment, depending on the stage of the tumor and the interaction between the stroma and epithelium. Raposo et al. showed that CD10 knockdown in the primary colon cancer cell line SW480 can increase the ability of these cells to migrate and invade. CD10 immunostaining studies have also shown an association between the advanced Duke stage and the presence of lymph node metastases (14).

The majority of adenocarcinomas of the colon are built of high cylindrical cells, similar to the dysplastic epithelium observed in adenocarcinomas. In most normal tissue, CD10 is expressed mainly in the apical part of the epithelium, mainly due to the involvement of healthy tissue in the secretory processes. CD10 is an endopeptidase that regulates signaling, hydrolysis of various peptide hormones, and cell growth (15, 16). Similar observations were made by Koga et al. CD10 was present along the apical portion of the tumor. Additionally, the level of CD10 expression was directly associated with tumor progression (17).

**Photo 1:**
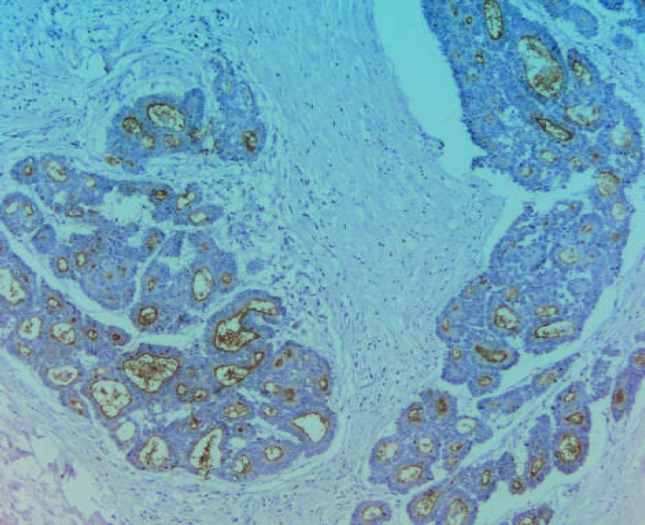
Immunohistochemical staining of denocarcinoma with profuse stroma. Focally in the apical parts of the cancerous tubes, expression of CD10 is visible. Magnification 100X

**Photo 2:**
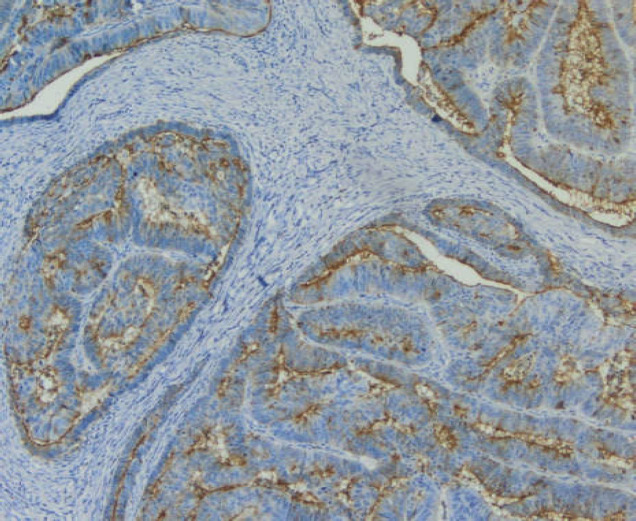
Foci of cancer cells comprising of irregular glandular tubes lined by high epithelium. Immunohistochemical staining of the cancerous cells reveals CD10 expression mainly in their apical parts. Magnification 400X

Many CD10+ cancers arise in tissues which express this protein even in the pre-cancerous state, suggesting that these cells probably retain some of their primary functions during tumorigenesis (18, 19, 20). In the current study, malignant cells showed clear expression of CD10 by immunohistochemistry, especially in their apical parts (Photos 1, 2). 

This may be related to the enzymatic activity of CD10. In new tumors, there is an accumulation of peptides cleaved by CD10, which affects the proliferation of undifferentiated cells. CD10 may also act by altering signaling pathways. It also inactivates physiologically diverse neuropeptides by cleaving peptide bonds at the amino terminus of hydrophobic amino acids. Thus, lower expression of CD10 could be responsible for tumor progression through the presence of peptide refurbishments for higher cell signaling in cancer, further facilitating tumor proliferation (21).

**Photo 3:**
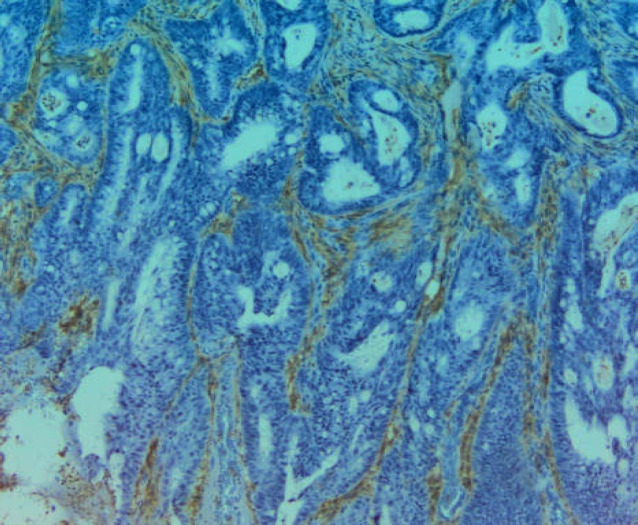
Immunohistochemical staining of CD10 in the stroma. Magnification 100X

**Figure 4:**
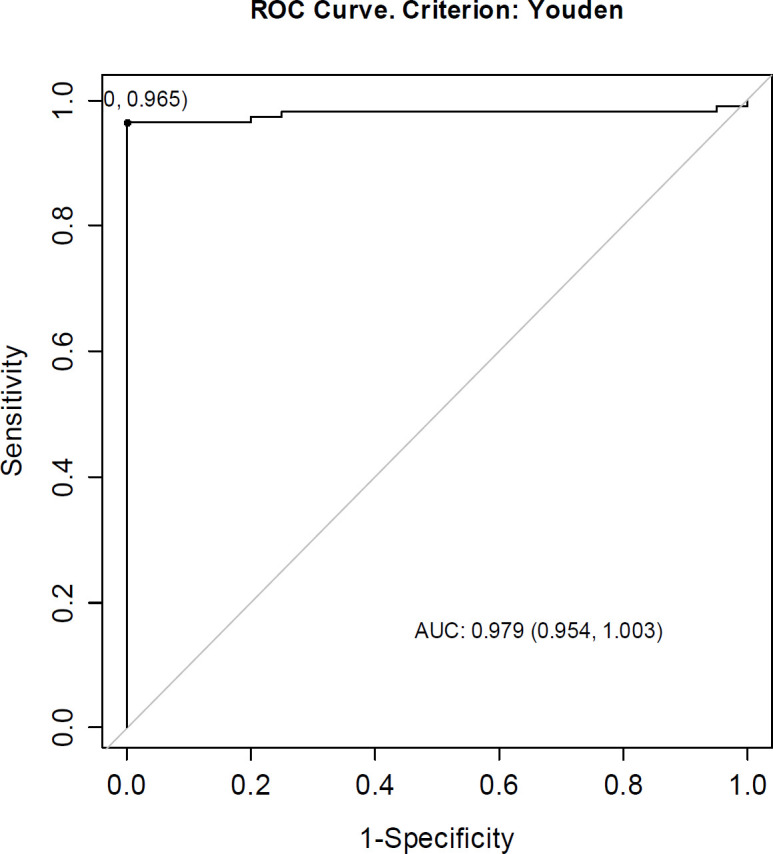
Receiver Operating Characteristic (ROC) curve for CD10 as a discriminator of study group (patients diagnosed with colorectal adenocarcinoma based on histopathological examination and treated) vs. control group (patients qualified for appendectomy)

CD10 is also useful in the diagnosis of microvillus atrophy. In healthy mucous, CD10 shows a linear pattern of staining (22), which is lost in patients diagnosed with colitis and Crohn’s disease (23). In cancers of the colon, immunohistochemical staining of CD10 reveals its presence not only in the cancerous cells but also in the stroma of the tumor (24, 25, 26). Similarly, in patients with pulmonary adenocarcinoma, stromal CD10 expression is an unfavorable prognostic factor related to an existing state of hypoxia (21).

In line with these findings, CD10 expression was also detected in the stroma of the tumor (Photo 3). In adenocarcinomas, CD10 expression is also seen in the cytoplasm of cells (27). The presence of CD10 in various cell regions may suggest its high cytophysiological activity in neoplastic cells. Oyama et al. found differences in CEA and CD10 staining between the conventional and clear cellular components. The CEA localization is associated with tumor differentiation. It is localized in the apical part of the lumen in well-differentiated tumors and the cytoplasm in poorly differentiated tumors. The diffuse cytoplasmic expression of CEA and the limited expression of CD10 observed in areas with bright cells may indicate that these components of the clear cells have a higher malignant potential (28).

CD10 expression in the serum and tissue of patients diagnosed with adenocarcinoma of the colon correlates with cancer staging based on the Astler-Coller-Dukes classification. CD10 could, therefore, be a useful marker for the early diagnosis of adenocarcinoma of the colon. The present study had a relatively small patient group (n = 113). Multicenter studies with a large number of patients may be able to help determine the reference serum CD10 levels, which could correlate with the histological grade of colorectal adenocarcinoma.

## Conflict of interests

The authors declare that they have no conflict of interest.
